# Do not discriminate only on age for cardiovascular risk management!

**DOI:** 10.1007/s12471-023-01840-w

**Published:** 2023-12-12

**Authors:** Fabrice M. A. C. Martens

**Affiliations:** Department of Cardiology, Amsterdam UMC—VU location, Amsterdam, The Netherlands

## Does cardiovascular risk management (CVRM) still have benefits in older patients?

In most of the larger cardiovascular studies the real elderly patients are not included. Also the possible therapy duration is shorter, while all cardiovascular (CV) risk reduction graphs show divergence in treatment time, regardless of the therapy, giving an increasing reduction in risk with an increased duration of treatment. Furthermore, the risk of side effects could be higher in older patients. So CVRM in older patients has been debated for decades.

However, mainly the elderly (75–95 years) suffer from cardiovascular disease and there is substantial evidence in pharmacological as well as in Mendelian randomisation studies that ‘the longer/earlier the lower LDL cholesterol, the better it is, despite which treatment regime’ [1]. The most recent meta-analysis by the Cholesterol Treatment Trialists’ Collaboration showed that even patients aged > 75 years have benefit [2] without side effects on cognition [3].

Strict blood pressure control in the elderly was even more of a dispute for years because of the known U‑shape curve in cardiovascular benefits and the obvious side effects when the blood pressure is too low. However, after for example the publication by Lewington et al. [4] the idea arose that also ‘the longer/earlier the lower (asymptomatic) blood pressure, the better it is’ especially in the elderly. In addition, there even seems to be a protective role for CVRM against, for example, vascular dementia [5], but evidence for this is still poor.

*Thus CVRM in older patients still has benefits, but we have to take frailty, life expectancy, expected therapy benefit and therapy harm into account.*

## Does more aggressive CVRM have enhanced benefits in older patients with ischaemic heart disease?

The higher the baseline cardiovascular risk and the more aggressive the CVRM, the greater the therapy benefit that could be expected. However, therapy benefit is also positively related to the possible duration of CVRM, which is often much shorter in older patients. In addition, competing risk is also a bias to be taken into account, especially in the elderly. Furthermore, the risk of side effects could be higher in older patients so, again, we have to take frailty, life expectancy, expected therapy benefit and therapy harm into account.

Van Trier and her team nicely estimated from the PHARMO Database Network (which links primary and secondary healthcare settings), using the SMART-REACH model, that in 1817 patients aged 71–80 years and hospitalised for ischaemic heart disease (36% women; median age at event of 74 years), the additional effect of a low-density lipoprotein cholesterol (LDL-c) target of < 1.4 mmol/l instead of < 1.8 mmol/l and a blood pressure target of < 130 mm Hg instead of < 140 mm Hg was still a median gain of 0.6 event-free life years [6]. As expected, the greatest effect (up to 1.7 event-free years) could be achieved in patients not even reaching the less strict targets. Do not forget that not only the statistical calculation is an estimation, but also the SMART-REACH model uses estimated data from several large randomised trials. What is worrying is the considerable number of patients with missing risk factor documentation (1186 of the 3003) in the study by Van Trier et al., suggesting that CVRM in older patients is no longer taken very seriously.

As already shown in the SPRINT trial, targeting a blood pressure to < 120 mm Hg versus < 140 mm Hg resulted in a hazard ratio of 0.75 in 9361 very high-risk CV patients, 28% of whom were older than 75 years (mean age 79 years) without more clinically relevant side effects [7].

For LDL‑c lowering, it has been extensively proven (pharmacologically as well as with Mendelian randomisation) that there is a log-linear association per unit change [1].

*Thus more strict CVRM in older patients still has benefits, especially when a cardiovascular event has already occurred (except in patients with end-stage heart failure or on dialysis), but we always have to take frailty, life expectancy, expected therapy benefit and therapy harm into account.*

## How do we define an ‘older’ patient? And in the future?

We have to be aware that elderly patients are often defined as aged over 70 years, but nowadays this is more an average old age while ageing is an ongoing process. We still lack robust evidence for the real elderly aged > 80 years.

## Does the Dutch CVRM guideline differ very much from the European (ESC) Prevention guideline?

Last but not least I would like to address that in the study by Van Trier et al. there is a controversial separation between the most recent Dutch CVRM guidelines of 2019 and the most recent ESC Prevention Guidelines of 2021 due to different LDL‑c and blood pressure targets. However, in the Dutch guideline the targets are set at *at least* < 1.8 mmol/l for LDL‑c and *at least* < 140 mm Hg for blood pressure, which means that in the Netherlands we can aim for European targets as well. Furthermore, in the ESC guideline the ‘Two Step Approach’ is introduced, in which the first step of CVRM targets of LDL‑c lowering of < 1.8 mmol/l and for blood pressure < 140 mm Hg have to be aimed for in all (very) high-risk CV patients, while a more aggressive approach with targets of LDL‑c lowering < 1.4 mmol/l and for blood pressure < 130 mm Hg should to be aimed for if thought necessary and according to the lifetime CV risk and treatment benefits, comorbidities, frailty and patient preferences.

## References

[1] Ference BA, Ginsberg HN, Graham I (2017). Low-density lipoproteins cause atherosclerotic cardiovascular disease. Evidence from genetic, epidemiologic, and clinical studies. A consensus statement from the European Atherosclerosis Society Consensus Panel. Eur Heart J.

[2] Gencer B, Marston NA, Im K (2020). Efficacy and safety of lowering LDL cholesterol in older patients: a systemic review and meta-analysis of randomized controlled trials. Lancet.

[3] Samaras K, Makkar SR, Crawford JD (2019). Effects of Statins on Memory, Cognition, and Brain Volume in the Elderly. J Am Coll Cardiol.

[4] Lewington S, Clarke R, Qizilbash N (2002). for the Prospective Studies Collaboration. Age-specific relevance of usual blood pressure to vascular mortality: a meta-analysis of individual data for one million adults in 61 prospective studies. Lancet.

[5] Tadic M, Cuspidi C, Hering D (2016). Hypertension and cognitive dysfunction in elderly: blood pressure management for this global burden. BMC Cardiovasc Disord.

[6] Van Trier T, Snaterse M, Herings RM (2024). Impact of implementing Dutch vs. European guideline risk factors in older patients with ischaemic heart disease. Neth Heart J.

[7] Wright JT, Williamson JD, Whelton PK (2015). for the SPRINT Research Group. A Randomized Trial of Intensive versus Standard Blood-Pressure Control. N Engl J Med.

